# Red Colour of Rhubarb with Alkalies

**Published:** 1811-06

**Authors:** 


*To the Editors of the Medical and Physical Journal.*

Gentlemen,

I SHALL feel much obliged to any of your chemical readers for the explanation of the red colour which immediately results from the combination of rhubarb with the carbonate of potash, or any of the other alkalies.

Murray, in his Elements of Materia Medica, states the constituents of rhubarb to be gum, resin, gallic acid, and a quantity of earthy matter, chiefly lime, combined with the sulphuric and citric acids.

By the insertion of the above you will much oblige an old Correspondent. Your's, &c.

APOTHECARIUS.

*Ipswich, March* 24, 1811.

